# Inside the Michelangelo effect: The role of art and aesthetic attractiveness on perceived fatigue and hand kinematics in virtual painting

**DOI:** 10.1002/pchj.606

**Published:** 2022-09-28

**Authors:** Marco Iosa, Fabiano Bini, Franco Marinozzi, Gabriella Antonucci, Simona Pascucci, Giulia Baghini, Valeria Guarino, Stefano Paolucci, Giovanni Morone, Gaetano Tieri

**Affiliations:** ^1^ Department of Psychology Sapienza University of Rome Rome Italy; ^2^ IRCCS Fondazione Santa Lucia Rome Italy; ^3^ Department of Mechanical and Aerospace Engineering Sapienza University of Rome Rome Italy; ^4^ Department of Life, Health and Environmental Sciences University of L'Aquila L'Aquila Italy; ^5^ Virtual Reality Lab Unitelma Sapienza of Rome Rome Italy

**Keywords:** aesthetics, art, kinematic, neuroaesthetics, neurorehabilitation, paint

## Abstract

It has recently been discovered that during a virtual reality task of painting, if the subjects have the illusion of recreating an artistic masterpiece, they improve their performances and perceive less fatigue compared to simply coloring a virtual canvas. This phenomenon has been called the *Michelangelo effect*. However, it was unclear if this effect was related to the aesthetic experience of beauty or if it was specific to artistic stimuli. To clarify this point, 26 healthy subjects performed the virtual task of erasing a blank sheet on the canvas, revealing an image that could be a painting or a photo, classified as beautiful or not. Beautiful paintings were famous artistic portraits, non‐beautiful paintings were rough reproductions of them. Photos of popular people were matched with paintings according to their similarity for somatic traits, posture, and clothes. Beautiful and non‐beautiful photos were classified according to whether the pictured person was famous or not for their beauty. For each stimulus the objective beauty, subjective beauty, and effort to complete the task perceived by the subject were self‐assessed on a numerical rating scale, recorded and analyzed. Furthermore, the hand kinematic trajectory was instrumentally recorded and its spatiotemporal parameters were computed. Less fatigue was perceived for the paintings than for the photos (*p =* .020), but not for beautiful versus non‐beautiful stimuli (*p =* .325). Only in the artistic stimuli, subjective beauty was found to be negatively correlated with perceived fatigue (*p =* .030) and performed errors (*p =* .005). The kinematic parameters were found to be affected by the interactions between the gender of the participant and that of the person in the photo. These results supported the idea that the Michelangelo effect was stronger when subjects interacted with artefacts, modulated by the perceived beauty of the artistic stimulus.

## INTRODUCTION

Artistic masterpieces feed into a general feeling of pleasure, motivation, and brain arousal: most observers of art works are familiar with feelings of empathetic engagement with what they see in the work itself (Freedberg & Gallese, 2007). There is the activation of wide brain areas during the observation of an art masterpiece, even including the motor cortex. It was hypothesized that it is due to the activation of mirror neurons when the spectator is observing depicted people performing actions (Freedberg & Gallese, 2007) and/or to the mental process of recognition of the represented motor intentions (Adolphs et al., 2000). However, similar wide activations were found in abstract art, conceivably because of the observer's implicit imagination of the artist's gestures that generated the traces on the canvas (Knoblich, 2002; Umiltà et al., 2012). It was also shown that the empathy produced by art on the observers could be strictly intertwined with the aesthetical judgement of the paint, especially for non‐expert people (Ardizzi et al., 2021).

Recently, an immersive virtual reality system was used to induce the illusion on healthy subjects and neurological patients of painting famous masterpieces. Subjects wore a head‐mounted display for virtual reality holding in their preferred hand the controller joystick, which represents a brushing sphere allowing them to color a white canvas on an easel in the virtual environment. Moving the brush on the canvas, they could unveil a famous artistic masterpiece (covered by a thin white layer that the brush deleted) or simply color the canvas (control condition). Even though the motor task required was the same in both conditions (the hand should be moved on the entire canvas to unveil the artwork or to color the canvas), the perceived fatigue and the erroneous movements orthogonally to the canvas were lower when subjects interacted with art (Iosa et al., 2021). This phenomenon of an improvement of subjects' performance in the presence of an artistic stimulus has been called the Michelangelo effect. The use of virtual reality gave the subject the illusion of painting on a canvas. The virtual task was developed warranting to the participants the senses of presence, embodiment, and agency, increasing motivation and engagement (Sanchez‐Vives & Slater, 2005; Tieri et al., 2018). Even though the experience of being in front of an authentic art masterpiece cannot be replaced in terms of explicit hedonic attributed values by virtual reproductions, it has been shown that faithful high‐quality virtual artwork reproductions could be as arousing as the original stimuli (Siri et al., 2018).

In the neuroaesthetic field, only a few studies have compared artistic stimuli with photos representing a similar subject. Ardizzi and colleagues compared artworks and photos representing painful faces finding similar brain activations (Ardizzi et al., 2021). Di Dio et al. (2011) compared the observations of artistic sculpture pictures and photos of young athletes with similar body postures, proportion, and expressed dynamism. The two stimulus categories produced rather similar global cortical activation patterns, but the right antero‐dorsal insula was more activated during sculpture viewing only. This is a region that does not merely mediate emotions but links emotion to cognition (Di Dio et al., 2011). Chirico and colleagues reported that in a virtual 360° scene, the nature‐based environment created more feelings of a sense of presence, fear, positive affect, and perception of the sublime than art‐based stimuli (Chirico et al., 2021).

The aim of the present study was to clarify whether the Michelangelo effect is specific to artistic stimuli, or whether it depends on the beauty of the stimuli.

## METHODS

### Participants

A sample of 26 healthy adults (23 ± 3 years old, 13 females), without any master degree education related to art, were enrolled in this study.

### Virtual reality procedure

The procedure was the same as in the previous study which revealed the Michelangelo effect (Iosa et al., 2021). Each subject sat wearing an Oculus Rift head‐mounted display and holding in their preferred hand the Oculus Controller joystick, which allowed them to interact with the virtual stimuli. The virtual environment was designed using 3ds MAX 2018 and implemented in Unity 2018 game engine software. It consisted of a large and comfortable room in which there was a white canvas (60 cm × 40 cm) on an easel. The subject interacted with the canvas with a virtual painting sphere, displayed in virtual reality in the same place of their real hand grasping the Oculus Controller. Subjects had been previously instructed that the sphere could color the canvas when in contact with it, forming a painting. The illusion of painting was given thanks to a thin white virtual panel (1200 pixels, each one with an area of 2 cm^2^) placed in front of the canvas (60 cm × 40 cm = 2400 cm^2^) which occluded the visibility of an underlying image. When the subject touched a few pixels of the virtual panel, they were automatically erased allowing you to see a part of the picture below. To overcome the lack of tactile information related to the actual touch of the virtual canvas, we included visual feedback. The color of the sphere changed from grey to green when the virtual sphere touched the canvas, colliding with the panel's pixels, or becoming red if an erroneous movement was performed beyond the canvas plane. Before the experiment, each participant underwent a calibration task. Then, participants were asked to color the canvas roughly starting from its center and then leaving them free to color the entire canvas following a self‐selected trajectory. The performance of the subject was recorded, tracking the position of the virtual sphere/real hand in space.

### Stimuli

**FIGURE 1:**
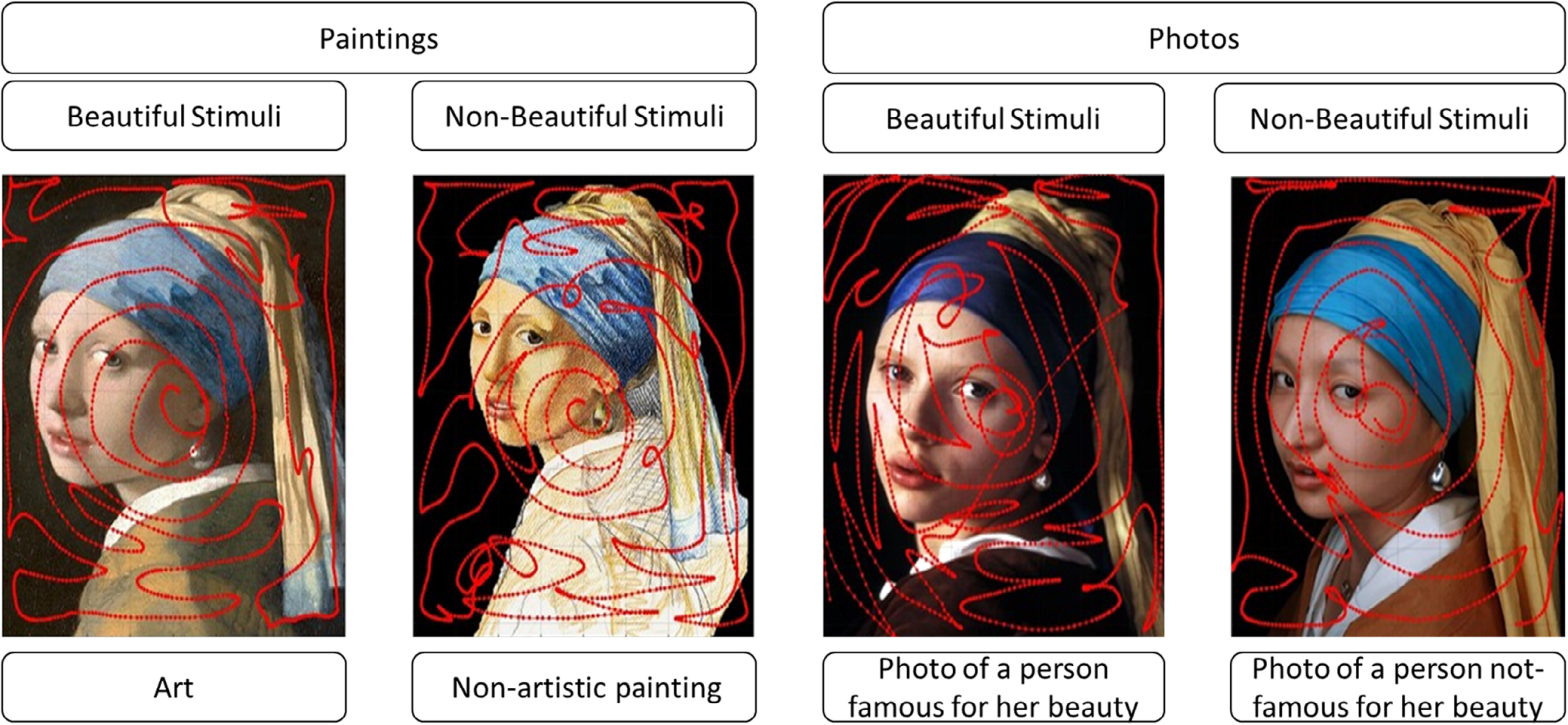
The example of four stimuli for *The Girl with a Pearl Earring* by Vermeer, with superimposed trajectory of the hand of one subject used to unveil the stimuli

Each subject interacted in a random order with 32 stimuli, divided into eight blocks of four types. The four types were: (1) a famous artistic portrait; (2) an artefact that was a rough painted copy of the art masterpiece; (3) a photo of a person famous for their beauty similar to the artistic portrait for somatic traits, posture and clothes; (4) an analogous photo of a person famous but not for their beauty. Table 1 reports the 32 stimuli divided between paintings and photos, beautiful and non‐beautiful stimuli. Figure 1 presents an example of the four types for one of the eight portraits.

**TABLE 1:** The stimuli: Four portraits focused on the portrait of a female (above) and those on the portrait of a male (below)

Paintings		Photos	
Beautiful	Non‐beautiful	Beautiful	Non‐beautiful
*The Birth of Venus* by Botticelli (1486)	Rough reproduction	Nicole Kidman standing with red hair	Meryl Streep standing with red hair
*The Mona Lisa* by Leonardo da Vinci (1506)	Pencil reproduction	Monica Bellucci with dark dress and crossed hands	Melissa McCarthy with dark dress and crossed hands
*The Girl with a Pearl Earring* by Vermeer (1665)	Pencil reproduction	Scarlett Johansson in the movie Girl with a Pearl Earring	He Yuhong dressed as the Girl with a Pearl Earring
*The Kiss* by Klimt (1908)	Rough reproduction	Megan Fox with a golden dress	Frida Khalo with a golden scarf
Adam of *The Creation of Adam* by Michelangelo (1512)	Rough reproduction	David Gandy lying in underwear	Sacha Baron Cohen lying in underwear
The Christ of *The Last Judgement* by Michelangelo (1541)	Rough reproduction	Chris Evans without shirt	Adam Sandler without shirt
*The Desperate Man* by Courbet (1845)	Rough reproduction	Johnny Depp with a hand behind the head	Joe Black with his hands close to his head
*Self‐Portrait with Beard* by Van Gogh (1889, Musée d'Orsay)	Pencil reproduction	Chris Hemsworth with beard	Ron Howard with beard

### Psychophysics and kinematic assessment

The perceived fatigue and the errors accomplished by the subjects (in terms of hand positions out of the canvas) were the main outcomes investigated in relation to the effects of the type of portrait (painting vs. photo) and the beauty of it. After each of the 32 trials, subjects were verbally asked to judge on a numerical rating scale ranging from 0 (*not at all*) to 10 (*the maximum possible*): how objectively beautiful the picture was (objective beauty), how much they liked the stimulus (subjective beauty), and how tiring the exercise was (perceived fatigue). All subjects were Italians and the original questions were hence in Italian (“Quanto reputa oggettivamente bella l'opera?,” “Quanto è bella l'opera per lei?,” and “Quanto è stato faticoso l'esercizio?”)

The virtual reality system allowed the registration of the hand position during each trial, as shown in Figure 1. The following kinematic parameters were extracted and analyzed: time to complete the task, length of the trajectory, jerk along the three axes (*z*‐axis was orthogonal to the canvas), entropy, and the errors evaluated as the number of frames in which the subject had their hand out of the canvas surface. The jerk is a parameter related to the fluidity of movements and it has been evaluated along the three axes as the derivate of acceleration integrated with respect to time and normalized for the length and duration of the trial (Yan et al., 2000). Being the stimulus on a planar surface, the jerk along *z*‐axis (jerk‐*z*) represented the non‐functional movements. Entropy is an estimation of the complexity of the trajectory, and hence it can be considered inversely related to the efficacy of movements performed to complete the task. According to previous studies, entropy was computed as the logarithm of the double of trajectory length divided by its convex hull (Cordier et al., 1994; Mendès France, 1981; Seifert et al., 2014), which in our task was confined into the perimeter of the canvas.

### Statistical analysis

All analyses were performed using IBM SPSS Statistics Version 23.0 (Armonk, New York). The sample size was chosen in line with previous results (Iosa et al., 2021). The data are reported in terms of mean ± standard deviation. Mixed analysis of variance was used to analyze the main factors, both within‐subjects (main factors computing collapsing other factors: beauty vs. non‐beauty, paintings vs. photos, gender of the person represented in the stimulus) and between‐subjects (gender of the subject performing the experiment), together with their interactions. To take into account the within‐subject variability also for the correlation analysis, we assessed the Pearson's coefficient (R) on the z‐scores of each variable. *Z*‐scores were assessed as the measured value subtracted by the mean computed among the four types of each stimulus, divided by the relevant standard deviation. All the analyses were tested with two‐tails and the alpha level of statistical significance was fixed at .05; post‐hoc analysis of mixed analysis of variance was used for which the Bonferroni's correction was applied according to the level of interaction.

## RESULTS

### Psychophysics results

**FIGURE 2:**
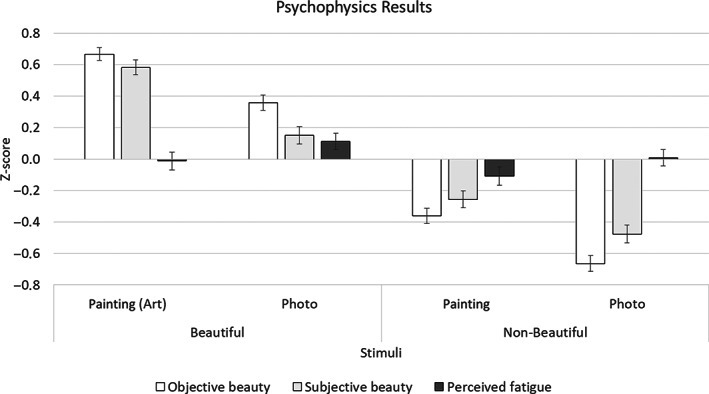
Psychometric results: *z*‐score ± standard error of self‐assessed on a numeric rating scale of objective (white bars) and subjective (light grey bars) beauty and perceived fatigue (dark grey bars) for the four types of stimuli

The analysis of self‐reported perceptions (shown in Figure 2 in terms of *z*‐score) confirmed the hypothesized categorization of the stimuli in terms of beauty. In fact, both beauty and subjective pleasantness resulted significantly higher in stimuli categorized as beautiful (effect of beauty factor as main factor collapsing paintings and photos: *F*(1,204) = 293.96, *p <* .001; *F*(1,204) = 179.89, *p <*.001, respectively). Even *The Desperate Man* by Courbet, which was judged as the least beautiful painting, scored higher than the non‐artistic paintings (7.77 ± 1.27 vs. 6.69 ± 1.72, *p =* .002). In general, the aesthetic judgements were higher for the paintings than for the relevant photos (main factor, collapsing beauty and non‐beauty: *F*(1,204) = 35.96, *p <* .001; *F*(1,204) = 27.43, *p <* .001, respectively). Objective and subjective beauty resulted higher also for stimuli representing females (*F*(1,204) = 27, *p <* .001; *F*(1,204) = 34.90, *p <* .001). Pleasantness was scored higher by female than by male participants (*F*(1,204) = 5.82, *p =* .017), but without any correlation with the gender of the subject represented in the portrait (*p =* .472).

As shown in Figure 2, the perceived fatigue generally resulted lower for the paintings than for photos (*F*[1,204] = 5.46, *p =* .020). Male participants scored higher in terms of fatigue compared to women (*F*[1,204] = 5.19, *p =* .024). Perception of fatigue was neither affected by beauty (*F*[1,204] = 0.97, *p =* .325) nor by interactions of other factors with beauty. Post‐hoc analyses showed non‐significant differences between artistic and non‐artistic paintings (*p =* .017).

### Kinematic results

Lower entropy (and hence a less complex trajectory) was associated with paintings than with photos (*F*(1,204) = 3.96, *p =* .048). No other variables significantly influenced the complexity of the trajectory.

The jerk‐z result was affected by the interaction of beauty with the observer's gender (*F*[1,204] = 4.19, *p =* .042). Women showed a significant lower jerk in the presence of a beautiful stimulus (post‐hoc: *p =* .020), whereas no differences were observed in men (*p =* .363). This jerk result was affected also by the interaction of picture type with the stimulus's gender (*F*[1,204] = 8.20, *p =* .005) with lower jerks for the paintings representing males than for those representing females (*p <* .001), whereas no gender differences were found for the photos (*p =* .232). These interactions also significantly affected the jerk along *x*‐ and *y*‐axes (*p <* .05).

The errors, assessed as frames spent out of the canvas, were affected by the interactions between the type of stimulus and the represented gender (*F*[1,204] = 5.29, *p =* .022). In fact, fewer errors were performed for photos of females than for paintings of females (*p =* .008), a difference not found when the subject of the portrait was a male (*p =* .994).

The length of trajectory and time spent to complete the task were neither affected by the analyzed main factors nor by their interactions.

### Correlations between variables

In the following, only statistically significant correlations are reported for each of the four types of stimuli.

For the artistic paintings, slight but statistically significant correlations were found for subjective beauty with perceived fatigue (*r* = –.151, *p =* .030), and with performed errors (*r* = −.194, *p =* .005).

For photos of beautiful people, perceived fatigue was related to the length of trajectory (*r* = .194, *p =* .005). Entropy was significantly correlated with the fatigue perceived for beautiful photos (*r* = .194, *p =* .005). The jerk‐*z* and the errors were both significantly correlated with the length of trajectory and the duration of the task (*r* > .135, *p <* .05).

For non‐beautiful paintings, the jerk‐z was significantly correlated with subjective beauty (*r* = .149, *p =* .032), perceived fatigue (*r* = –.222, *p =* .001), error (*r* = .232, *p =* .001), and duration (*r* = .580, *p <* .001). Errors and fatigue were neither related to objective nor to subjective beauty.

For other photos, the perceived fatigue was correlated with the jerk‐z (*r* = –.178, *p =* .010). The jerk‐z and errors were correlated each other (*r* = .189, *p =* .006), and both with the time spent to complete the task (*r* = .553, *p <* .001, *R* = .145, *p =* .037, respectively).

## DISCUSSION

The aim of this study was to assess if the Michelangelo effect (Iosa et al., 2021), the reduction of perceived fatigue and performed errors during the interaction with artistic stimuli in a virtual task, was specific for art or related to the beauty of the stimulus. In order to answer to this research question, we arbitrarily selected artistic portraits and compared them to non‐artistic paintings and photographic portraits of people famous or not for their beauty, roughly matching body postures, colors, clothes, facial expressions, and somatic traits. First of all, the categorization of the selected stimuli was confirmed by the aesthetic judgements of beauty and pleasantness provided by the participants. The need for differentiating between objective and subjective beauty was related to potential different neural circuits related to these two aesthetical judgements (Di Dio et al., 2007).

Our main result was that the perceived fatigue was lower when subjects unveiled the paintings with respect to the photos. Also, aesthetical judgements were higher for the paintings than the photos. The main effect of beauty was not statistically significant, but a significant correlation between subjective beauty and perceived fatigue was found for artworks and not for the other stimuli. These psychometric results were supported by kinematic analysis. In fact, entropy of movements, which was an estimation of the complexity of trajectory, was lower for paintings than for photos. Finally, hand trajectory was more fluid (lower jerk) for beautiful than for non‐beautiful stimuli, but only for female participants.

Hence, beauty seemed to act not as a main factor, but as a modulator reducing the perceived fatigue and the errors in artistic paintings. The effect of beauty in non‐artistic paintings seemed to be indirect: more pleasant paintings had higher jerks, related to smaller fatigue and errors. This relationship could be interpreted as the perception of fatigue related to the execution of complex movements required for improving the precision of hand movements in contact with the virtual canvas. This idea is confirmed by the results of the complexity of the trajectory quantified by the entropy of the hand movements (Cordier et al., 1994). This complexity was lower in paintings than in photos, like the perceived fatigue. Furthermore, in beautiful photos, the greater the complexity of the trajectory, the greater was the perceived fatigue.

Our results are quite different from those of Chirico et al. (2021), who reported stronger feelings in the observers of virtual scenes when they were obtained by natural photos with respect to those obtained by artistic paintings. An important difference was that our stimuli were paintings or photos of humans, whereas in that study the stimuli were about scenery. Furthermore, unlike their study (and most neuroaesthetic studies), in our virtual task the participant was not a mere observer: the participant was asked to progressively unveil the stimulus. So, in our protocol, the perception of beauty could be strictly intertwined with the actions performed to actively unveil the stimulus. The subject could be considered at the same time the producer and the observer of the stimulus. This approach is partially in line with the “functional parallelism theory” proposing that the artist and the visual brain of the observers are driven by the same visual goal and purposes: to organize visual information in order to capture the essential features of the scene and produce a faithful representation of them (Zeki, 1999). This functional parallelism was criticized for the differences among neuroaesthetics, aesthetic perception, art knowledge, and artistic techniques to produce visual meaning effects in the artworks (Bundgaard, 2015). On the other hand, the ecological affordances included in the stimulus may lead to its own aesthetic attractiveness (De Bartolo et al., 2021). Furthermore, beauty, or the feeling of pleasure, is not an essential property of aesthetic experience related to visual art (Bundgaard, 2015). *Guernica* by Picasso and *The Scream* by Munch are examples of visual artworks that are not strictly related to the concept of beauty. A recent study also showed that graffiti murals were classified as art to a lesser extent than abstract paintings, but subjects liked the murals more than the abstract paintings, suggesting that art classification might not necessarily predict perceived beauty (Szubielska & Ho, 2021). In our study, we used artistic portraits, most of them associated with a feeling of beauty, as confirmed by the psychophysics results reported in Table 1. We did not test art not associated to an aesthetical experience of beauty, but we investigated the effect of beauty not associated to art, such as in photos of beautiful people.

In our results, the main effect seemed to be played by the type of portrait: paintings vs. photos. Scientific literature reports that the aesthetic judgements were highly reliable and conserved across individuals for natural stimuli, such as photos of landscapes (Bignardi et al., 2021) or human faces (Vessel et al., 2018). Conversely, the aesthetic judgements of artefacts of human culture seemed to be less reliable, lacking uniform behavioral relevance for most individuals, conceivably because this is based on more individual aesthetic sensibilities, reflecting varying experiences and different sources of information. However, in our study, the virtual reality task required participants to depict an image on canvas, and in this context it could be more ecological to paint a painting than a photo. For example, Zhang and Zeki (2022) classified equations for mathematicians as biological stimuli, given their ecological meaning for them, and tested the reliability of their aesthetic judgements related to equations. They found a high reliability even in presence of a confounding fictitious expert rating, similarly to that of natural stimuli.

But not only differences between paintings and photos were noted. Beauty seemed to act as a modulator of the performance, as well as the gender of the depicted subject. Only for artistic paintings, perceived fatigue and performed errors were inversely correlated with the subjective perception of beauty.

The kinematic errors were not dependent on the type of stimuli, but on gender‐related factors. Female participants reported lower fatigue than males, especially for stimuli judged more beautiful. This finding could be read in the light of a recent study reporting that the virtual reality treatment was more effective in female than male patients (Bruschetta et al., 2022). The authors hypothesized that it was due to the strongest relationship between exercise and cognition in females, but it could also be due to gender differences in artistic perceptions.

The main limits of our study were related to the use of a virtual task. Further studies should investigate the possible presence of the Michelangelo effect in front of art in the real world and not only in the virtual context, or in front of artworks not directly associated to the concept of beauty (such as *Guernica* or *The Scream*). It should be kept in mind that the tested virtual reality protocol was designed for neurorehabilitation of patients with upper limb deficits for improving their motivation and their participation (Iosa et al., 2021). With this study, we investigated if the specific effect found for art was due to beauty or to the use of artefacts. Further studies could also include an analysis of where the subjects were looking on the canvas. De Bartolo and colleagues reported that subjects are often focused on specific regions of interest related to the harmony of the picture, an aspect that may link the aesthetical judgement with an objective criterion, such as the relative proportions of the depicted figure (De Bartolo et al., 2021). Another potential limit of our study is that the difference in the results may at least be partially due to individual differences between participants, which has not been verified up front, for example personality, cultural level, or artistic tastes.

In conclusion, we found a reduction of perceived fatigue and errors when subjects interacted with the paintings, with a further reduction in the case of beautiful stimuli, suggesting that the Michelangelo effect is powerful in artistic masterpieces inducing pleasure to the observers, but that this effect could also depend on the individual feeling of beauty associated with the work and also on what is represented in the scene.

## CONFLICT OF INTEREST

The authors declare no potential conflicts of interest with respect to the research, authorship, or publication of this article.

## ETHICS STATEMENT

This study was approved by the Independent Ethical Committee of Santa Lucia Foundation. Each participants signed the informed consent.
